# RECRUITING COMMUNITY-DWELLING LIVE-ALONE PERSONS WITH DEMENTIA: AN EXPLORATION OF FIVE GATEKEEPER DOMAINS

**DOI:** 10.1093/geroni/igz038.3522

**Published:** 2019-11-08

**Authors:** Laura Girling, Kate de Medeiros

**Affiliations:** 1 University of Maryland, Baltimore County, Baltimore, Maryland, United States; 2 Miami University, Oxford, Ohio, United States

## Abstract

Although recruiting persons with dementia into research is challenging enough, finding those who live-alone in the community is even more difficult. Consequently, live-alone persons with dementia are often overlooked and/or deliberately excluded from inquiry despite calls for more inclusive approaches to dementia research. Based on enrollment strategies from an interview-based protocol recruiting 120 live-alone persons with dementia, our National Institute on Aging- funded study identified five domains of gatekeepers imperative to gaining access to community-dwelling, live-alone persons with dementia: 1) housing (e.g., service coordinators), 2) data proprietors (e.g., regulatory specialists), 3) institutional (e.g., review boards), 4) kin (including fictive kin), 5) clinical (e.g., medical providers, clinician practices). In addition, gatekeeper domains are multilayered and serve distinct roles in both facilitating and hindering access to and enrollment of this under-researched vulnerable population. Analysis of our recruitment efforts contribute significant insights into how the dementia research community may engage the various domains of community gatekeepers, providing direction for current and future social science research.

